# An ERP Study of the Temporal Course of Gender–Color Stroop Effect

**DOI:** 10.3389/fpsyg.2020.613196

**Published:** 2021-01-13

**Authors:** Yingli Li, Juan Du, Qingfang Song, Sina Wu, Lihong Liu

**Affiliations:** ^1^Department of Psychology, Soochow University, Suzhou, China; ^2^Department of Applied Human Sciences, Western Kentucky University, Bowling Green, KY, United States; ^3^School of Chinese Language and Literature, Beijing Foreign Studies University, Beijing, China; ^4^Department of Psychology, Guangdong University of Foreign Studies, Guangzhou, China

**Keywords:** gender stereotype, gender–color interference, pink, blue, Stroop effect, ERP

## Abstract

Pink and blue colors have been found to associate with gender stereotypes in previous Western studies. The purpose of the present study was to explore the neuropsychological processing basis of this effect in contemporary Chinese society. We presented stereotypically masculine or feminine occupation words in either pink or blue colors to Chinese college students in a modified Stroop paradigm, in which participants were asked to classify each occupation word by gender as quickly and accurately as possible. Event-related potential (ERP) signals were concurrently recorded in order to identify the temporal dynamics of gender stereotypical interference effect. The behavioral results showed that pink–masculine stimuli elicited a longer response time and lower accuracy than blue–masculine stimuli in the participants, while no such differences were observed between pink–feminine and blue–feminine conditions. The ERP results further revealed distinctive neural processing stages for pink–masculine stimuli (i.e., in comparison to the other three types of stimuli) in P200, N300, N400, and P600. Overall, our results suggested that pink but not blue was a “gendered” color in Chinese culture. Moreover, our ERP findings contributed to the understanding of the neural mechanism underlying the processing of gender–color stereotypes.

## Introduction

Gender stereotypes are behavioral and personality characteristics of different genders expected or required by people (Eagly and Steffen, 1984). As one of the most common stereotypes, gender stereotype involves expectations of certain colors associated with different genders. Specifically, in Western culture, girls are often dressed in pink, while boys are often dressed in blue (Pomerleau et al., 1990). These gender–color associations have emerged in the Western world since the 1950s (Frassanito and Pettorini, 2008; Ben-Zeev and Dennehy, 2014; Grisard, 2018), and such color preference differences between the two genders may extend from childhood to adulthood (e.g., Cunningham and Macrae, 2011). Nevertheless, a recent study demonstrated, at least in some Western societies, that pink is a color restricted to female gender (i.e., only girls prefer pink), whereas blue is a neutral color that is commonly favored by both genders; such female gender preference toward pink was found in children as young as 10 years old (Jonauskaite et al., 2019).

Gender-associated colors not only are related to fashion business but also function as symbols embodied in gender stereotypes. Some researchers have proposed that gender is one of the most salient social categories providing powerful heuristic for structuring incoming social information (Most et al., 2007). As such, gender-associated color symbol is an important tool for people to judge a person’s gender in a time- and energy-saving way. Moreover, gender-associated color symbols may reinforce gender stereotype-consistent thinking and behaviors. For example, several studies have demonstrated that gender-associated colors (i.e., pink vs. blue) can activate participants’ gender stereotypical thinking, leading them to behave in a manner more consistent with gender stereotypes (Cunningham and Macrae, 2011; Yeung and Wong, 2018).

The Stroop task is deemed as one of the best task tapping on the difficulty people have in processing two conflictual features of a stimulus. In the classic Stroop test (Stroop, 1935), a Stroop effect is revealed by participants’ longer reaction time and lower accuracy for naming color words with inconsistent printed color (e.g., word “blue” in red ink) than those with consistent printed color (e.g., word “blue” in blue ink). Several recent studies have adapted the Stroop task to examine gender stereotypical conflicts. Specifically, gender stereotypical names (e.g., Rachel) or objects (e.g., football) were spoken by either a feminine or a masculine voice (e.g., Green and Barber, 1981; Most et al., 2007). Stroop effect was observed whereby participants took longer to determine the sex of the voice when the spoken word was associated with its stereotypically opposite sex (e.g., “football” spoken by a feminine voice). Nevertheless, there is only one study that has examined the Stroop effect of color-primed gender stereotypical conflicts (Cunningham and Macrae, 2011). In their experiments (3–5), different stimuli (i.e., names, sex-typed objects, or faces of different genders) were presented in either pink or blue color, and participants were asked to classify the gender of the stimuli as quickly as possible. The results showed that the response time for categorizing color-matching conditions (e.g., female name in pink ink or male name in blue ink) was significantly shorter than that for the color-mismatching conditions (e.g., male name in pink ink or female name in blue ink), suggesting that pink and blue function as gender symbols.

Despite that the symbolic association of pink and blue colors with different genders has been established in the literature, most of the previous studies use response time and accuracy rates to reflect participants’ cognitive performance. The neural mechanism underlying the cognitive processing of gender stereotypes primed by color remains obscure. As proposed by some researchers (e.g., Bartholow, 2010), event-related potentials (ERPs) can provide a direct and time-sensitive assessment of such cognitive processes. The technique of ERP is known for its high temporal resolution and therefore suitable for examining brain activation changes over milliseconds (Luck and Kappenman, 2011). Importantly, ERP components can reflect collective activation of neural units engaged in specific cognitive processes over a time course.

Event-related potential has been used to examine gender stereotypical conflicts. For example, participants are presented with a gender pronoun following a target word that has gender stereotypical implications. Word pairs that are incongruent in gender association (e.g., “nurse-he,” “driver-she”) elicited different electrophysiological activities from the congruent word pairs (e.g., “driver-he,” “nurse-she”; White et al., 2009; Irmen et al., 2010; Siyanova-Chanturia et al., 2010; Wang et al., 2017). Different patterns of electrophysical activities are observed in two particular ERP components, N400 and P600, which, respectively, reflect the processing of semantic information (e.g., Kutas and Federmeier, 2000) and gender stereotype violation (e.g., Osterhout et al., 1997). Overall, ERP has been seen as an effective approach to reveal the complex cognitive processes involving gender stereotypes.

While previous ERP studies mainly used gender pronoun as a prime to activate gender category, no ERP study has used word color as a prime of gender stereotypes and examine how our neural system processes the incongruence of gender stereotypical information implied in word color and word meaning. In addition, majority of those studies (except for Wang et al., 2017) were conducted in Western societies, and little is known about whether similar gender–color association exists in contemporary Chinese society. Frassanito and Pettorini (2008) have pointed out that gender-defined application of pink and blue is relatively exclusive to Western culture. Moreover, many studies suggest that there is no conclusive knowledge about the fixed gender–color preferences across different cultures (Jadva et al., 2010; Paoletti, 2012; Wong and Hines, 2015; Zhan and Dan, 2015; Jonauskaite et al., 2019).Therefore, it is worthy to explore how pink and blue are gender-symbolically represented by Chinese people.

The present study aimed to explore the neural activation pattern underlying the parallel processing of color-associated and word meaning-associated gender stereotypical information in Chinese adults. To this end, a modified word–color Stroop task was utilized, in which gendered occupation words were presented in either blue or pink colors, and participants were asked to classify the occupation words into different genders as quickly and as accurately as possible. A group of Chinese college students participated in the study, and their brain activity was recorded and analyzed using the technique of ERP.

Several ERP components (i.e., P200, N300, N400, and P600) of particular importance to color and gender stereotypical information processing would be examined in our study. Specifically, increased P200 amplitude has been found to relate to enhanced automatic attention in early time windows (Lu et al., 2010), as well as stimulus evaluation and incongruence detection (Chen et al., 2009). Shortened P200 latency has also been observed in processing incongruent or negative stimulus (Gan et al., 2016). Increased N300 amplitude has been found to accompany the integration of color with other information such as shape or word (Bramão et al., 2012; Gan et al., 2016), as well as inhibition of unrelated interference attributes (Xiao et al., 2009). Color processing has been found to result in variation in N300 latency (Gan et al., 2016). Increased N400 amplitude has been found to associate with long-term memory information integration (Kutas and Federmeier, 2000, 2011), as well as conflicting stereotype information processing (Wicha et al., 2004; White et al., 2009; Chen and Yu, 2015). Variation in N400 latency has also been observed in gender-related language information processing (Proverbio et al., 2010; Wang et al., 2017). Finally, increased P600 amplitude has been found to relate to syntactic violation detection (Jia et al., 2010; Wang et al., 2010) and implicit stereotype conflict processing (Lattner and Friederici, 2003; Wicha et al., 2004). Value processing related to words has been found to cause variation in P600 latency (Gan et al., 2016). Notably, stereotypical conflict processing may significantly involve the functioning of several brain regions, including frontal (Amodio, 2014) and parietal lobes (Adleman et al., 2002), and may have a leftward inclination (Proverbio et al., 2017). Based on the literature, it was hypothesized that our behavioral data would reveal a gender stereotypical interference effect, that is, participants would show a longer reaction time and lower accuracy in incongruent conditions (i.e., pink–masculine and blue–feminine) than congruent conditions (i.e., blue–masculine and pink–feminine). Moreover, it was expected that incongruent stimuli would provoke different activation patterns from those of congruent stimuli in the ERP components of P200, N300, N400, and P600 (i.e., evidenced in both strengthened amplitude and shortened latency).

## Materials and Methods

### Participants

A total of 30 Chinese undergraduate students (17 females, *M*_*age*_ = 19.90 years, *SD* = 0.55) participated in this experiment on a voluntary basis. Given that the Stroop test usually involves a large effect size (for a meta-analysis, see Verhaeghen and De Meersman, 1998), the sample size of the present study ensured *a priori* power of 0.90 for our repeated-measures ANOVA design according to the calculation of G-Power (Faul et al., 2007). The input parameters were: repeated ANOVA, *f* = 0.25, α = 0.05, number of groups = 1, repetition = 4, power = 0.90, correlation among repeated measures = 0.5, and non-sphericity correction = 1.

All participants were right-handed, with normal or corrected-to-normal visual acuity and no history of neurological diseases or color blindness (i.e., measured by Ishihara test), based on a physical examination conducted at the beginning of college entrance. Ethical approval for the study was obtained from the Psychology Department at Soochow University, and all participants signed a consent form.

### Materials

Twenty Chinese words representing stereotypically masculine jobs (e.g., *repairer*) and 20 Chinese words representing stereotypically feminine jobs (e.g., *secretary*) were adapted from previous studies (Siyanova-Chanturia et al., 2010, 2015). These job words were judged by a separate group of college students (*n* = 10) as appropriate for their gender categories. All words consisted of two or three characters (see Table 1 for details).

**TABLE 1:** Masculine and feminine job categories.

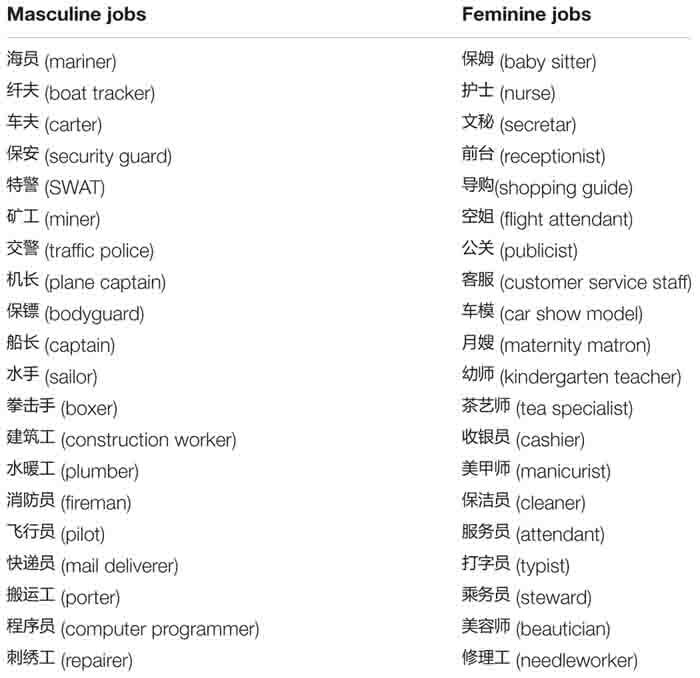

### Procedure

The present experiment followed a 2 (Word Gender: masculine vs. feminine) × 2 (Color: pink vs. blue) repeated-measures ANOVA design.

All participants completed the experiment on the same computer in an EEG lab, which was an enclosed soundproof space, lit by a 100-lux ceiling lamp.

**FIGURE 1:**
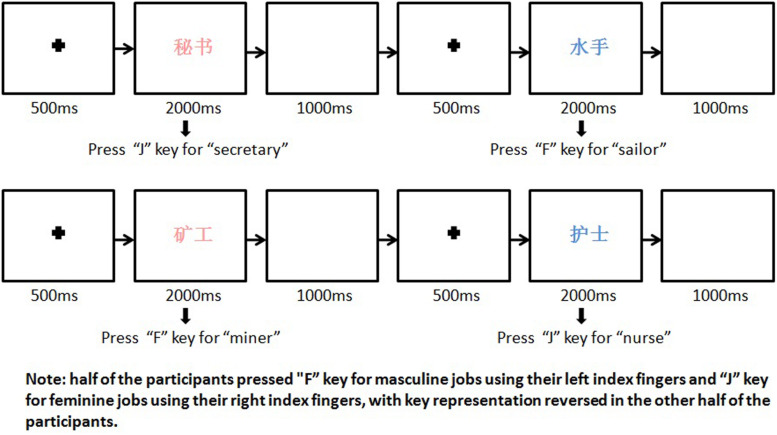
Sample stimulus presentation setting.

Participants were familiarized with the experiment *via* a practice session (six trials) and were required to repeat practicing until they reached 100% accuracy. During the formal test, each job word was presented in 72-point SimSun system font, with a vertical visual angle of 0.5° and a horizontal visual angle of 1° (for two-character words) or 1.02° (for three-character words). Following the classic Stroop test paradigm, each job word was presented in either pink or blue (RGB_*pink*_ = 255,192,203; RGB_*blue*_ = 0,0,255; Cunningham and Macrae, 2011; Al-Rasheed, 2015), resulting in 400 trials in total (i.e., 100 trials for each Word Gender × Color condition). All stimuli were programmed *via* E-prime 2.0 (Psychology Software Tools, Inc.) and shown in a random sequence on a 27-inch computer screen with a white background (Figure 1). For each trial, following a fixed black cross (500 ms), a target occupation word was presented in the center of the screen for a maximum time of 2,000 ms (participants were required to respond within 2,000 ms). The between-trial interval was 1,000 ms. Participants were allowed to take a short break (30–60 s) after every 100 trials. Participants were instructed to classify each occupation word by gender as quickly and accurately as possible. Half of the participants were asked to press “F” key for masculine jobs using their left index fingers and “J” key for feminine jobs using their right index fingers. Key representation was reversed for the other half of the participants (i.e., “J” for masculine jobs and “F” for feminine jobs). Reaction time (i.e., interval between stimulus presentation and response) and accuracy (i.e., percentage of masculine and feminine occupation words being correctly classified into their designed gender categories) were recorded.

### EEG Recording and Data Preprocessing

EEG data were recorded concurrently during the formal test *via* a 64-channel cap (Brain Products, Munich, Germany). All electrodes were arranged according to the international 10–20 system. Horizontal electro-oculogram (HEOG) and vertical electro-oculogram (VEOG) were also recorded. Electrode impedances were maintained below 5 KΩ, with a sampling rate of 1,000 Hz and a 0.01–35-Hz band-pass filter. The use of a 0.01–35-Hz band-pass filter in our study is a conventional choice, as many people believe that neural activities above 35 Hz do not arise from the brain and likely represent noise or artifacts (Libenson, 2009). EEG data were offline analyzed *via* the software ANALYZER 2.0. The original EEG data were re-referenced to the average of whole brain and then corrected for eye-blink artifacts (i.e., epochs with EEG power exceeding ± 100 microvolt). Afterward, the artifact-free data were segmented into 1,000-ms post-stimulus EEG epochs (baseline corrected with a 200-ms pre-stimulus interval) and then averaged for each stimulus type (i.e., masculine occupation words in pink, masculine occupation words in blue, feminine occupation words in pink, and feminine occupation word in blue). Following the convention (Libenson, 2009), peak amplitudes and latencies of P200 (150∼250 ms), N300 (250∼350 ms), N400 (350∼450 ms), and P600 (550∼650 ms) over 20 electrode sites (i.e., F1, F2, F3, F4, and Fz over the frontal area; FC1, FC2, FC3, FC4, and FCz over the frontal-central area; C1, C2, C3, C4, and Cz over the central area; P1, P2, P3, P4, and Pz over the parietal area) were extracted for statistical analysis.

## Results

A preliminary analysis showed no significant gender differences in either the behavioral or the ERP data. Therefore, gender was not used as a factor for the following analyses.

### Behavioral Results

Means and SDs for behavioral response on the Stroop test were shown in Table 2.

**TABLE 2:** Mean and SD for accuracy and reaction time on gender-color stroop test.

		**Accuracy**		**Reaction time**	
		**M(%)**	**SD**	**M(ms)**	**SD**
Male participants	Blue-masculine	98.92	1.89	690.3	80.44
	Pink-masculine	97.15	3.72	714.27	78.29
	Blue-feminine	93.15	7.66	681.55	60.82
	Pink-feminine	93.69	5.47	681.9	71.7
Female participants	Blue-masculine	97.65	2.03	656.93	93.93
	Pink-masculine	96.21	4.07	679.8	93.65
	Blue-feminine	90.94	9.67	678.62	88.11
	Pink-feminine	93.14	8.24	656.28	75.97
Total	Blue-masculine	98.20	2.04	671.39	88.47
	Pink-masculine	96.62	3.89	694.74	87.62
	Blue-feminine	91.90	8.78	679.89	76.26
	Pink-feminine	93.38	7.06	667.38	74.02

Reaction time data for trials with incorrect response or unreasonable duration (i.e., ±3 *SD* deviated from the mean) were removed. The remaining 95.12% of the original data were analyzed *via* a 2 (Word Gender: masculine vs. feminine) × 2 (Color: pink vs. blue) repeated-measures ANOVA. The two main effects were not significant: *F_Word Gender_* (1, 29) = 1.322, *p* = 0.260, *partial eta^2^* = 0.044; *F*_*Color*_ (1, 29) = 1.168, *p* = 0.289, and *partial eta^2^* = 0.039. However, a significant Word Gender × Color interaction effect was found: *F* (1, 29) = 13.34, *p* = 0.001, *partial eta^2^* = 0.32. Follow-up paired *t* tests showed that, while participants responded significantly faster to blue–masculine stimuli than pink–masculine stimuli [*t* (29) = 3.31, *p* = 0.003], no such difference was found between blue–feminine and pink–feminine conditions [*t* (29) = 1.79, *p* = 0.084]. An additional paired *t* test showed that reaction time for pink–feminine stimuli was also significantly shorter than that for pink–masculine stimuli [*t* (29) = 3.64, *p* = 0.001].

For accuracy, the 2 (Word Gender) × 2 (Color) repeated-measures ANOVA showed a significant main effect for Gender [*F* (1, 29) = 10.03, *p* = 0.004, *partial eta^2^* = 0.257], with the overall accuracy on masculine occupation words being higher than that on feminine occupation words. The main effect of Color was not significant: *F* (1, 29) = 0.01, *p* = 0.914, and *partial eta^2^* = 0.000. Importantly, the Word Gender × Color interaction effect was significant: *F* (1, 29) = 6.69, *p* = 0.015, and *partial eta^2^* = 0.19. Follow-up paired *t* tests showed that, while the accuracy of blue–masculine stimuli was higher than that of pink–masculine stimuli [*t* (29) = 2.56, *p* = 0.016], no such difference was found between those of blue–feminine and pink–feminine stimuli [*t* (29) = 1.66, *p* = 0.109].

These results showed that, despite that both blue–feminine and pink–masculine trials were considered incongruent conditions, a Stroop effect was only found for pink–masculine trials.

### Event-Related Potential Results

In the behavioral results, a Stroop effect was found only in pink–masculine condition. Therefore, our EPR data analysis would be focused on revealing distinct neural activation patterns for this Stroop effect by using Type (pink–masculine, blue–masculine, pink–feminine, and blue–feminine) as the independent factor. Electrodes were organized by two topographic factors: Mediality (left, left-middle, middle, right- middle, and right) and Longitude (frontal, frontal-central, central, and parietal). Peak amplitudes and latencies of P200, N300, N400, and P600 were analyzed *via* 4 (Type) × 4 (Mediality) × 5 (Longitude) repeated-measures ANOVAs. For simplicity, only significant results involving the factor of Type would be reported (i.e., significant results involving only topographic factors are generally considered as irrelevant).

**FIGURE 2:**
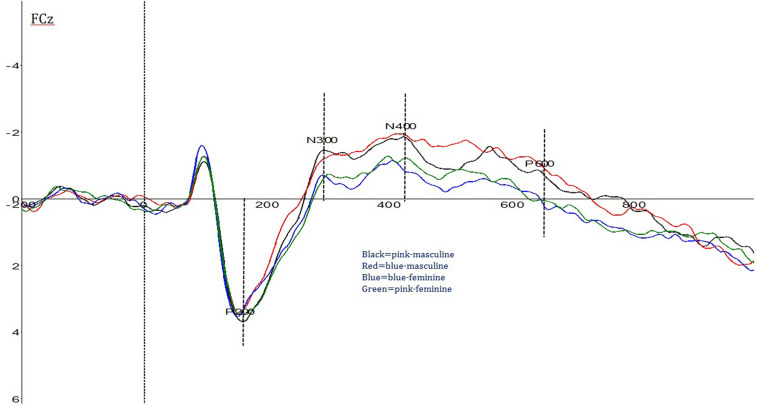
Overall event-related potential (ERP) activation patterns for the four stimulus types.

**FIGURE 3:**
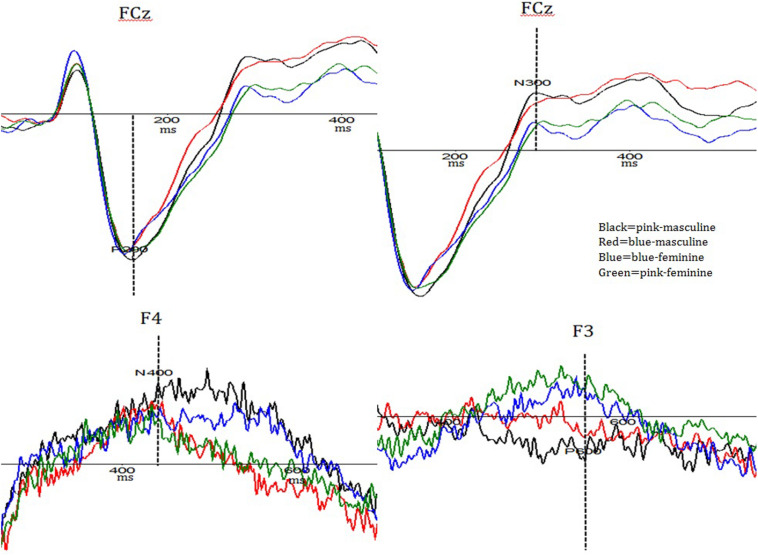
Component activation comparison among the four stimulus types.

Activation differences among the four types of stimuli were shown in Figures 2, 3.

#### Amplitude

For N300 amplitude, a significant main effect of Type was found: *F* (3, 87) = 2.94, *p* = 0.092, and *partial eta^2^* = 0.09. Overall, pink–masculine trials induced the largest N300 amplitude (*M* = −3.52_μ *V*_, *SD* = 1.25).

For **N400** amplitude, a three-way interaction effect of Type × Mediality × Longitude was found significant: *F* (36, 1,044) = 2.00, *p* < 0.001, and *partial eta^2^* = 0.07. In comparison to other types, pink–masculine trials elicited the largest N400 amplitude over the right frontal electrodes (*M* = −3.37_μ*V*_, *SD* = 1.95).

For P600 amplitude, a significant Type × Mediality interaction effect was found: *F* (12, 348) = 3.92, *p* < 0.001, and *partial eta^2^* = 0.12. In comparison to the other types, pink–masculine trials induced the largest P600 amplitude (*M* = 2.80_μ *V*_, *SD* = 1.73) over the left frontal electrodes.

#### Latency

For P200 latency, a significant main effect of Type was found: *F* (3, 87) = 4.88, *p* = 0.004, and *partial eta^2^* = 0.14. Overall, pink–masculine trials induced the shortest P200 latency (*M* = 196.24_*ms*_, *SD* = 12.02).

For N400 latency, a significant Type × Longitude interaction effect was found: [*F* (9, 261) = 2.68, *p* < 0.001, and *partial eta^2^* = 0.13]. Pink–masculine trials induced the shortest N400 latency over the frontal area (*M* = 374.66_*ms*_, *SD* = 35.10). Type × Mediality interaction was also significant: [*F* (12, 348) = 11.60, *p* < 0.001, and *partial eta^2^* = 0.29]. In comparison to the other types, pink–masculine trials induced the shortest N400 latency over the left frontal electrodes (*M* = 357.89_*ms*_, *SD* = 25.00).

For P600 latency, Type × Mediality interaction effect was found significant: *F* (12, 348) = 9.38, *p* < 0.001, and *partial eta^2^* = 0.24. In comparison to the other types, pink–masculine trials elicited the shortest P600 latency over the left and left-middle electrodes (*M* = 595.81_*ms*_, *SD* = 14.02).

## Discussion

The present study explored the temporal course of gender–color interference effect in Chinese college students by using ERP. Our hypotheses on the Stroop effect were only partially supported by the behavioral data. A gender–color interference effect was observed in pink–masculine condition but not in blue–feminine condition, suggesting that pink, but not blue, is a gender identity-related color in Chinese culture. Notably, participants in many Western societies usually treat pink as a feminine color and blue as a masculine color (Bargh, 1999; Macrae and Bodenhausen, 2000; Ben-Zeev and Dennehy, 2014). However, a recent study on Swiss participants’ color preference showed that, while pink was favored only by female participants, blue was favored equally by both genders, regardless of age (Jonauskaite et al., 2019). Our result is consistent with the latter, suggesting cultural variations on gender–color association, which may not simply conform to Eastern vs. Western division.

Our ERP data further revealed an interesting temporal course of gender–color interference processing for pink–masculine condition, which could be divided into the following four stages. In the first stage, pink–masculine trials induced the shortest P200 latency among the four types of trials. P200 latency has been found to relate to attention allocation and sensitive to negative visual stimuli (Lu et al., 2010; Leuthold et al., 2015). Shorter P200 latency for pink–masculine trials may suggest that this type of stimuli was more conspicuous than the other types of stimuli for our participants and therefore attracted quicker attention in early processing stage. In the second stage, pink–masculine trials induced the largest N300 amplitude. Previous studies have revealed that N300 amplitude is positively correlated to difficulty in integrating perceptual information with semantic meaning (Brianmcpherson and Holcomb, 2010; Bramão et al., 2012; Redmann et al., 2014; Gan et al., 2016). The largest N300 amplitude elicited by pink–masculine trials suggests that our participants needed extra effort to reconcile the conflicting gender information of “female” color (i.e., pink color) and “male” job (i.e., masculine occupation words). In the third stage, pink–masculine trials induced the largest N400 amplitude over the right frontal region as well as the shortest N400 latency over the left frontal region. N400 latency and amplitude have been demonstrated to be sensitive to the processing of semantic expectation violation as well as stereotype conflict, with severer violation or incongruence eliciting shorter N400 latency and higher N400 amplitude (Kutas and Federmeier, 2000; Wicha et al., 2004; White et al., 2009; Chen and Yu, 2015). Therefore, the largest and fastest N400 induced by pink–masculine trials may suggest that this type of stimuli strongly violated our participants’ expectations. In the fourth stage (P600), pink–masculine trials were found to elicit the largest amplitude over the left frontal region as well as the shortest latency over the left hemisphere. The literature has shown that P600 amplitude is related to the processings on syntactic violation (Jia et al., 2010; Wang et al., 2010) and implicit stereotype conflict (Lattner and Friederici, 2003; Wicha et al., 2004), while P600 latency is related to implicit evaluation of emotion-arousing events (Osterhout et al., 1997; Lattner and Friederici, 2003; Wicha et al., 2004; Jia et al., 2010; Wang et al., 2010). The largest and fastest P600 found for pink–masculine trials suggests that the combination of pink color with masculine identity may be highly expectation-violating and strongly emotion-arousing for our participants. Notably, this result is consistent with some previous studies involving Western participants, in which the image of a male dressed in pink were found to be highly aversion-arousing (Ben-Zeev and Dennehy, 2014) or stigma-triggering (Grisard, 2018).

In the present study, pink–masculine condition, but not blue–feminine condition, induced significant behavioral and ERP changes. Theoretically, both pink–masculine and blue–feminine stimuli were incongruent in nature and should show both behavioral and neural activation differences from the congruent stimuli (i.e., pink–feminine and blue–masculine). The unexpected differential behavioral and ERP results for pink–masculine and blue–feminine trials may be related to different implicit association strengths of gender–color pairs. In China, as well as many other societies, pink color may be strongly linked to feminine identity. In contrast, blue is not sternly restricted to masculine identity and, in fact, many women find blue color favorable (Ellis and Ficek, 2001; Al-Rasheed, 2015; Jonauskaite et al., 2019). Therefore, the association of pink–masculinity, but not the association of blue–femininity, is strongly anti-stereotypical and likely to produce significant behavioral and neural activation changes in our participants. Such finding is also consistent with stereotypical gender asymmetry, that is, people are more tolerant to women taking on masculine features than men taking on feminine features (e.g., Eagly and Steffen, 1984; Diekman and Eagly, 2000; Eagly et al., 2000).

Stereotypical gender–color associations are nurtured by society and culture (Ashmore and Del Boca, 1981) and can be viewed as “culture in mind” guiding the thinking of group members in a culture (Hinton, 2017). Future studies exploring how the stereotypical gender–color associations gradually develop in children and shape their neural activity may contribute to a deeper understanding of human brain plasticity (Rippon et al., 2014) and gender role development (Siyanova-Chanturia et al., 2015).

A few limitations in the present study are noteworthy. First, in the present study, gender implications of colors were measured implicitly. Despite that implicit measures are often thought as ecologically valid (Proverbio et al., 2017), a comparison of ERP results regarding implicit and explicit gender-associated colors may provide a richer picture for gender–color research field. Secondly, the sample size was relatively small and the gender distribution in the sample was not strictly balanced, which may potentially affect the generalizability of our results. Last but not the least, our study examined Chinese college students. We need to be cautious in generalizing the results to other populations, for example, adults who have lower than college education levels, who reside in rural areas, or those of different age groups. Studies involving participants from diverse backgrounds can help examine whether this effect would vary depending on different demographic factors.

In summary, it was found in the present study that the anti-stereotypical association of pink color with masculine identity (but not blue color with feminine identity) produced a significant Stroop effect and ERP changes over the temporal course. Four stages of pink–masculine information processing were identified. The first stage (P200) involved quick attention allocation to the unusual association of pink color with masculine identity. The second stage (N300) involved increased effort for integration of symbolic gender implication of pink color with masculine identity. The third stage (N400) and the fourth stage (P600) involved incongruence management and emotion evaluation related to pink–masculine association. Overall, our results suggested that pink but not blue was a “gendered” color for Chinese culture. These results not only supported the current findings on stereotypical gender–color associations but also contributed importantly to the understanding of the neural mechanism underlying the processing of gender–color stereotypes.

## Data Availability Statement

The raw data supporting the conclusions of this article will be made available by the authors, without undue reservation.

## Ethics Statement

The studies involving human participants were reviewed and approved by Ethics Committee of Soochow University (China). The patients/participants provided their written informed consent to participate in this study.

## Author Contributions

YL and JD were in charge of experimental design, data collection, data analysis, and manuscript writing. QS, SW, and LL participated in manuscript editing. All authors contributed to the article and approved the submitted version.

## Conflict of Interest

The authors declare that the research was conducted in the absence of any commercial or financial relationships that could be construed as a potential conflict of interest.
